# Non-ergodic fragmentation upon collision-induced activation of cysteine–water cluster cations†

**DOI:** 10.1039/d2cp04172c

**Published:** 2023-01-17

**Authors:** Lukas Tiefenthaler, Paul Scheier, Ewa Erdmann, Néstor F. Aguirre, Sergio Díaz-Tendero, Thomas F. M. Luxford, Jaroslav Kočišek

**Affiliations:** a Institute for Ion Physics and Applied Physics, University of Innsbruck Austria paul.scheier@uibk.ac.at; b Faculty of Applied Physics and Mathematics, Gdansk University of Technology Narutowicza 11/12 80-233 Gdansk Poland; c Departamento de Química, Universidad Autónoma de Madrid 28049 Madrid Spain sergio.diaztendero@uam.es; d Software for Chemistry and Materials (SCM) Amsterdam The Netherlands; e Condensed Matter Physics Center (IFIMAC), Universidad Autónoma de Madrid 28049 Madrid Spain; f Institute for Advanced Research in ChemicalSciences (IAdChem), Universidad Autónoma de Madrid 28049 Madrid Spain; g J. Heyrovský Institute of Physical Chemistry v.v.i., The Czech Academy of Sciences Dolejškova 3 18223 Prague Czechia jaroslav.kocisek@jh-inst.cas.cz

## Abstract

Cysteine–water cluster cations Cys(H_2_O)_3,6_^+^ and Cys(H_2_O)_3,6_H^+^ are assembled in He droplets and probed by tandem mass spectrometry with collision-induced activation. Benchmark experimental data for this biologically important system are complemented with theory to elucidate the details of the collision-induced activation process. Experimental energy thresholds for successive release of water are compared to water dissociation energies from DFT calculations showing that clusters do not only fragment exclusively by sequential emission of single water molecules but also by the release of small water clusters. Release of clustered water is observed also in the ADMP (atom centered density matrix propagation) molecular dynamics model of small Cys(H_2_O)_3_^+^ and Cys(H_2_O)_3_H^+^ clusters. For large clusters Cys(H_2_O)_6_^+^ and Cys(H_2_O)_6_H^+^ the less computationally demanding statistical Microcanonical Metropolis Monte–Carlo method (M_3_C) is used to model the experimental fragmentation patterns. We are able to detail the energy redistribution in clusters upon collision activation. In the present case, about two thirds of the collision energy redistribute *via* an ergodic process, while the remaining one third is transferred into a non-ergodic channel leading to ejection of a single water molecule from the cluster. In contrast to molecular fragmentation, which can be well described by statistical models, modelling of collision-induced activation of weakly bound clusters requires inclusion of non-ergodic processes.

## Abstract 2

Cysteine–water cluster ion fragmentation and internal energy partitioning upon collision-induced activation have been revealed by experiments with cluster ions assembled in He droplets and Microcanonical Metropolis Monte–Carlo M_3_C calculations.

## Graphical abstract

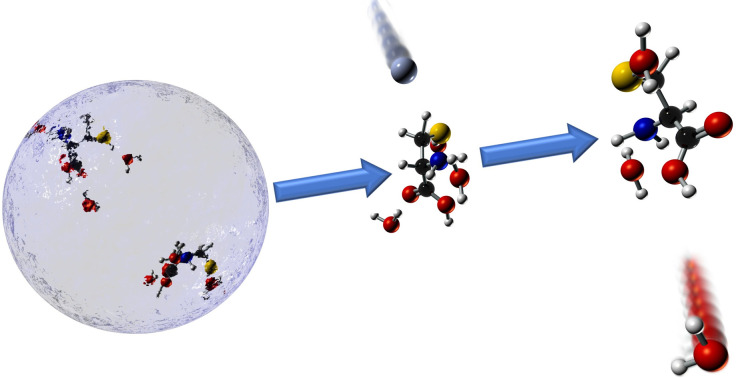

## 1 Introduction

Tandem mass spectrometry using collision-induced dissociation (CID)^1^ is a commonly used analytical technique.^2–4^ While fragmentation at a specific energy may provide a useful additional dimension to mass spectrometric analysis,^5,6^ it has also been applied to evaluate the thermodynamic parameters of the ion fragmentation processes and of ion-molecule reactions.^7,8^ Studied systems include *e.g.* isolated molecules, peptides, organometallic molecules or homogeneous clusters.^9–12^ For precise studies of the thermodynamics of a system, the initial energy distribution in the ion beam, the thermal energy distribution of the collision gas and kinetic and internal energies of the fragments must be known.^7,13^ In more complex systems, other parameters start to play an important role such as the inter-/intra-molecular relaxation processes.^14,15^ In clusters the relaxation processes, based on intermolecular energy transfer, complicate the evaluation of the data.^7^ For example, the clusters are able to absorb all the energy gained by collisional activation with no fragmentation on the timescale of the experiment, resulting in a so called kinetic shift of several eV in the determination of dissociation thresholds.^16^ True thresholds are then estimated based on the cross sections approximated by a power function with an adjustable exponent *N* characterizing the initial energy transfer process and transition state modelling,^17,18^ which assumes that the precursor ion is in its equilibrium state.^19^ The thermodynamic equilibrium assumption limits the usability of the method for processes slower than energy redistribution within the ion, and the *N* parameter then allows the extraction of correct numbers without knowing the details of the initial energy redistribution.

In the present work, we aim to shed more light on the initial energy redistribution process by combining CID experiments of cold Cys(H_2_O)_*n*_^+^ clusters with simulations based on density functional theory and statistical mechanics. Cys(H_2_O)_*n*_^+^ clusters represent an interesting biophysical and biochemical model from the point of view of fundamental metabolic processes in the human body and from the point of view of radiation biology of peptides. Cysteine is a nonessential amino acid but is present in several important biological processes. *E.g.* serum levels of cysteine were explored as an important marker for several diseases^20–23^ including many types of cancer^24–26^ and its metabolic pathways were recently discussed as an interesting target for cancer treatment.^27–29^ Cysteine is also a building block of the important antioxidant glutathione, with many applications from nutrition^30^ to cosmetics.^31^ A low reduction potential makes cysteine also interesting in the context of radiation-induced damage of living tissue^32–35^ and in biohybrid technology, where it has been shown to actively contribute to acetate and energy production.^36^

This all makes cysteine and its water complexes an interesting model system to study. Furthermore, an unusual zwitterionic form of this amino acid can be formed by deprotonation of the thiol group.^37,38^ Solvation of neutral cysteine was therefore explored by both theory and experiment.^39–43^ Isolated cysteine and fragmentation of its cation was theoretically explored by de Oliveira.^44^ Experimental studies of the isolated molecule include PEPICO,^45^ dissociative electron attachment^46,47^ or ion beam irradiation^48^ studies. However, it is important to say that studies of isolated cysteine are rather scarce due to difficulty to sublime this thermally delicate compound.^49^ An alternative approach is to study the molecules deposited on a surface, applied *e.g.* in the study of electron-induced desorption.^50^ Other approaches include atmospheric pressure ionization mass spectrometry (MS)^51^ and electrospray ionization MS where both positive and negative (deprotonated) ions of cysteine were studied.^52–56^ Here we prepared cysteine cations using a recently developed technique of cluster ion assembly inside He droplets^57^ allowing for measurements at very low sublimation temperatures of the sample. Combination of tandem mass spectrometry with simulations, carried out using density functional theory (DFT) and the Microcanonical Metropolis Monte–Carlo method (M_3_C),^58,59^ allows us to describe initial energy redistribution after activation of the Cys(H_2_O)_*n*_^+^ and Cys(H_2_O)_*n*_H^+^ ions by collisions with Ar atoms.

## 2 Methods

### 2.1 Experiments

**Fig. 1:**
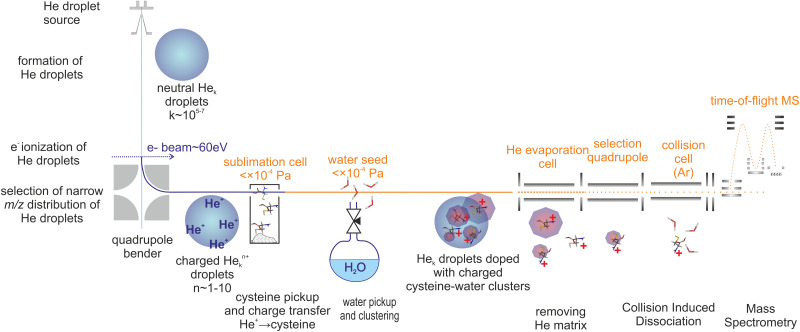
Schema of the experiment.

Cluster cations were prepared by ion assembly inside He droplets, a method recently developed in our laboratory.^57^ The CID experiment was performed using a modified tandem mass spectrometer (Waters Q-TOF Ultima). Fig. 1 is a sketch of the experiment.

Briefly, He droplets were prepared by He expansion at a stagnation pressure of 2 MPa through a 5 μm nozzle, and were kept at a temperature of 9 K. Droplets with an average size of about 10^6^ He atoms^60^ were multiply charged^61^ by electron ionization at an electron energy of ∼70 eV. The ionized droplets were deflected perpendicularly to the neutral droplet beam using an electrostatic quadrupole ion bender. The selected distribution of droplets bearing multiple^62^ He^+^ with respect to He_*n*_^+^ charge centers^63^ picked up cysteine vapour prepared by sublimation of a cysteine powder (Sigma-Aldrich 98%) in a resistively heated glass cell. Water was introduced into the same vacuum chamber at pressures in the 0.1 mPa range, allowing for assembly of cluster cations. Charge transfer to cysteine or water resulted in the formation of ion cores that were solvated with additional water molecules.

The mean number of water molecules can be controlled (see the ESI†). Several charged clusters are formed in each droplet.^61^ After pickup and charge transfer from He, the cluster ions inside He droplets are cooled down to a few kelvin.^63,64^ The helium droplets then pass through an evaporation cell filled with He at a constant pressure to shrink the droplets by multiple collisions with room temperature He gas. Coulombic repulsion between the charge centers increases during this process and leads to the sequential ejection of individual charge centers, including the charged cysteine–water clusters often still complexed with a few He atoms. The presence of He taggants to the cluster ions indicates a low temperature (determined by the binding energy of the weakest bound He atom) of the charged cluster.^65,66^

The released cluster ions were analyzed by means of CID. Details may be found in our recent paper.^65^ For CID, we used an Ar collision gas at a pressure of ∼1 mPa in a 9 cm long RF hexapole collision cell. The energy axis was calibrated using the retarding potential method.^13^ The intensity of the fragment ions is significantly lower than that of the precursor ions. This is quite a common observation,^66,67^ since fragment ions produced in the CID process occupy much larger phase space in comparison to precursor ions and therefore their transport and detection efficiency is lower. An additional complication of the present experiment is that our orthogonal TOF does not allow detection of ions at *m*/*z* < 40 so we cannot detect potentially formed H^+^ or CO^+^ fragment ions. The total ion signal in the CID measurements drops above 5 eV CM collision energy, which may be caused by the aforementioned fact. Therefore, we focus mainly on the part of the spectrum in the range of collision energies in the CM frame from 1 to 5 eV.

### 2.2 Computational details

Following our previous studies of fragmentation of isolated molecules,^68,69^ the theoretical approach applied in this work employs a two-step methodology considering energetic structures, time propagation and entropy maximization. First, the geometries of cysteine–water clusters involving three and six water molecules were optimized using the M06-2X functional^70^ and the 6-31++G(d,p) basis set^71–73^ of atomic orbitals. The choice of such a functional is rationalized by its effectiveness in calculating binding energies in systems with non-covalent interactions such as hydrogen-bonding.^74^ The initial geometries of neutral clusters have been obtained from a DFT study of microsolvated cysteine.^75^ The singly ionized molecules were obtained by removing one electron from the neutral system and optimizing the geometry. Additionally, protonated cysteine–water clusters (again involving three and six water molecules) have been obtained by the addition of one proton at the possible protonation sites (amine or thiol groups). For every geometry, an optimization calculation of harmonic frequencies was performed as a way to confirm that a true minimum was reached, *i.e.* no negative frequencies were obtained. The information obtained in the geometry optimization and in the frequency calculations is further employed in the fragmentation simulations (see below).

Second, for systems with three water molecules (both protonated and non-protonated) *ab initio* molecular dynamics simulations were carried out with the atom centered density matrix propagation (ADMP) method^76–78^ and the M06-2X functional^70^ combined with the 6-31G(d,p) basis set. The maximum propagation time was limited to 500 fs and a time step of 0.1 fs was chosen. The energies between 2 and 13 eV were deposited into the most stable isomer of the cysteine cation and protonated cysteine with three waters and randomly distributed over all nuclear degrees of freedom. For every energy, 25 trajectories have been calculated, giving together 225 trajectories for each system. Bond distances and charge distributions at the last dynamical step (500 fs) of every trajectory were indicators of reactive mechanisms with distances between atoms larger than *R* = 2.5 Å treated as broken bonds.

To treat fragmentation dynamics of large clusters such as Cys(H_2_O)_6_^+^ a computationally inexpensive method has to be used. Therefore, the Microcanonical Metropolis Monte Carlo method^58,59^ in its recent implementation in the M_3_C code^79^ was applied in the second step to calculate fragmentation branching ratios. In accordance with the ergodic theorem in the M_3_C method time averages are substituted with statistical space averages. As the main result the M_3_C method provides fragmentation probabilities as a function of the internal energy. The database of possible fragments was built from cysteine–water clusters optimized in the first step of our methodology (M06-2X/6-31++G(d,p) level of theory). Additionally, at the same level of theory it was necessary to optimize the water and protonated water clusters. The initial geometries of water^80^ and protonated water^81^ clusters for subsequent optimization were obtained from The Cambridge Cluster Database. The final number of species included in the M_3_C database is presented in the ESI.†

All quantum chemical calculations were performed with the use of the Gaussian09 software package.^82^

## 3 Results

**Fig. 2:**
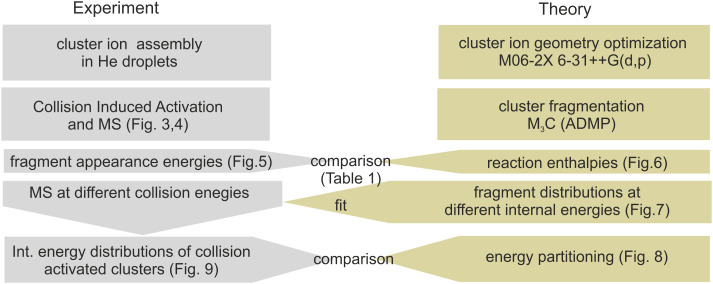
Overview of the obtained results and their interplay.

The experimental and computational steps performed in the present work are summarized in context in Fig. 2. This section starts with a presentation of the collision-induced activation mass spectra in subsection 3.1. We focus primarily on Cys(H_2_O)_6_H^+^ and Cys(H_2_O)_6_^+^ cations, while the supporting data for smaller clusters are in the ESI.† Subsection 3.2 is dedicated to a comparison of the experimentally measured appearance energies of individual fragmentation channels with the energies obtained from the theoretical models. Subsection 3.3 focuses on the energy partitioning after collisional activation based on the M_3_C model and fit to the experimental data.

### 3.1 Fragmentation MS upon CID

**Fig. 3:**
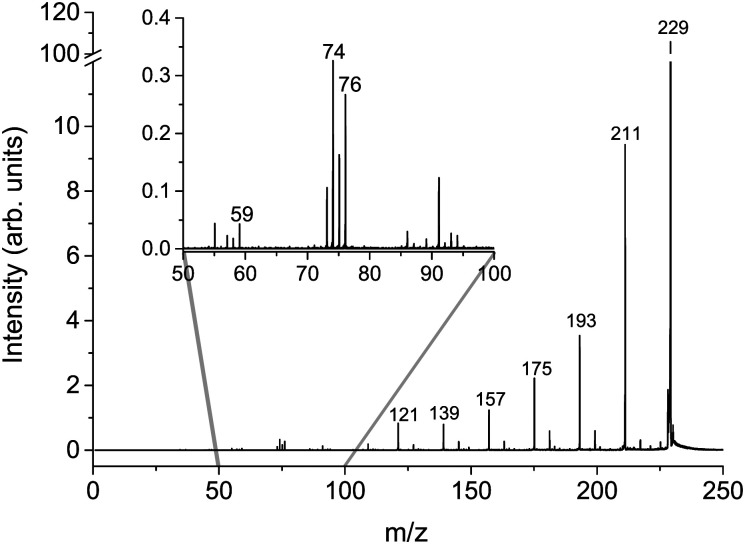
Cumulative MS for the *m*/*z* = 229 Cys(H_2_O)_6_^+^ precursor ion created as a sum of individual CID MS in counts per second obtained at lab frame energies of (8, 11, 16, 21, 26, 31, 36, 46, 56, 66, 76, 86, 96, and 106) eV.

**Fig. 4:**
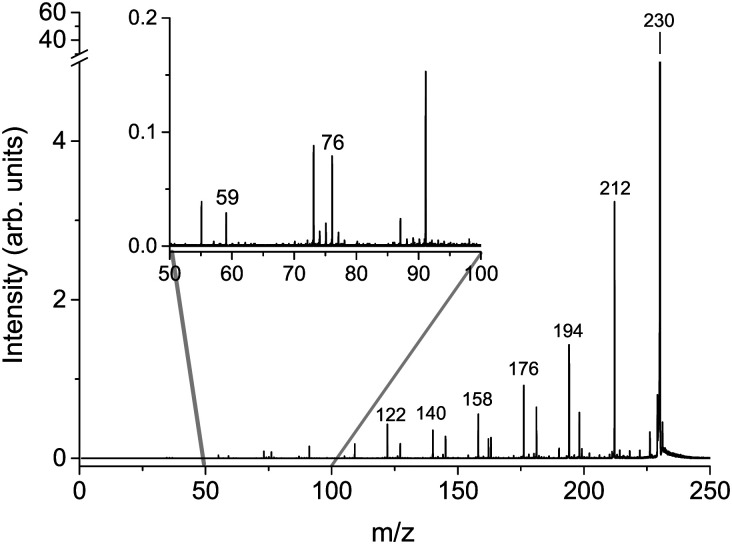
Cumulative MS for *m*/*z* = 230 Cys(H_2_O)_6_H^+^ precursor ion created as a sum of individual CID MS in counts per second obtained at lab frame energies of (8, 11, 16, 21, 26, 31, 36, 46, 56, 66, 76, 86, 96, and 106) eV.

The cumulative mass spectra in the collision energy range from 6 to 106 eV in the laboratory frame for Cys(H_2_O)_6_^+^*m*/*z* = 229 and Cys(H_2_O)_6_H^+^*m*/*z* = 230 cations can be seen in Fig. 3 and 4, respectively. The dominating ions in the mass spectra can be assigned to the loss of up to all six water molecules. In contrast to homogeneous amino acid cluster ions^66^ we do not observe a transition from the canonical to the protonated form. This means that the protonated form of the clusters Cys(H_2_O)_*n*−1_H^+^ does not result from a unimolecular decay of Cys(H_2_O)_*n*_^+^ clusters. They are rather the result of a proton transfer reaction at an early stage of the cluster assembly process.

In addition to the main fragmentation pattern, the spectra (Fig. 3 and 4) also contain a less intense progression of peaks resulting from the fragmentation of He_3_ (H_2_O)_12_H^+^ and He_8_ (H_2_O)_11_^+^, which have *m*/*z* overlapping with those of the precursor ions Cys(H_2_O)_6_H^+^ and Cys(H_2_O)_6_^+^, respectively. Avoiding clustering of cysteine and enhancing of water clustering at the same time, we end up at conditions where initial cluster distributions were dominated by water clusters (see the ESI†), causing this overlapping *m*/*z* contribution.

In the insets of the figures, we can see the main fragment ions of cysteine. The protonated form Cys(H_2_O)_6_H^+^ fragments mainly to the *m*/*z* = 76 H_6_C_2_NS^+^ fragment resulting from the loss of the carboxylic group and *m*/*z* = 59, which corresponds to C_2_H_3_S^+^ in agreement with previous studies.^52^ In the fragmentation pattern of the canonical form Cys(H_2_O)_6_^+^, we can see one more intense fragment at *m*/*z* = 74 corresponding to H_4_C_2_O_2_N^+^ resulting from the loss of CH_2_CSH. At low masses below *m*/*z* = 40, the spectrum is influenced by the transmission function of the instrument. For example, we are not able to detect an important fragmentation channel of protonated cysteine leading to the NH_4_^+^ cation.^83^ Because of this fact, our discussion of relative ion intensities in Subsection 3.3 will be restricted to fragmentation *via* evaporation of water. Fragmentation reactions of the embedded cysteine molecules will be discussed in the following Subsection 3.2 only in the context of their appearance energies.

### 3.2 Appearance energies

**Fig. 5:**
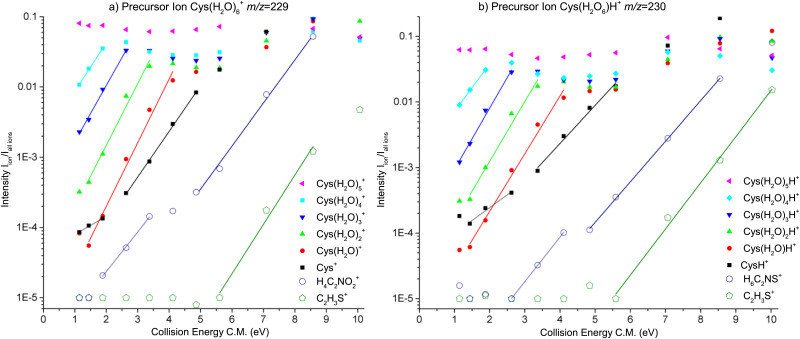
Relative ion yield curves for water loss and two most intense molecular dissociation fragments resulting from CID of canonical Cys(H_2_O)_6_^+^ or protonated Cys(H_2_O)_6_H^+^ clusters on Ar gas. The lines are linear fits to the data used for the estimation of dissociation thresholds.

**Fig. 6:**
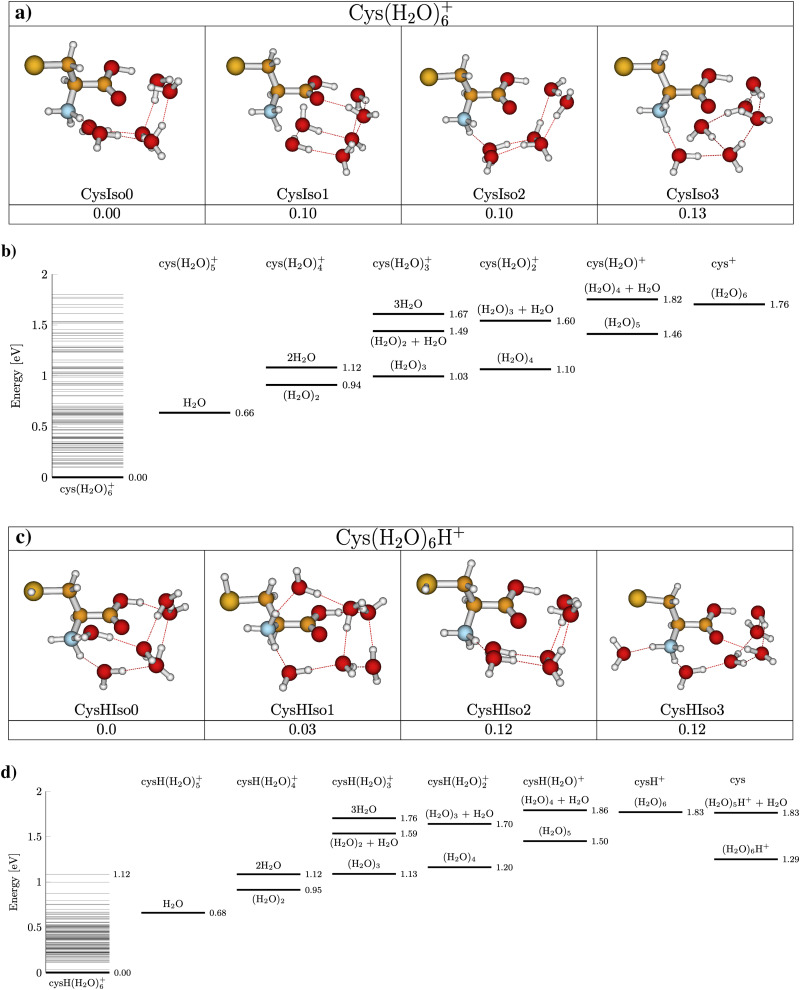
Most stable structures of Cys(H_2_O)_6_^+^ (panel (a)) and Cys(H_2_O)_6_H^+^ (panel (c)) cluster cations from M06-2X/6-31++G(d,p) calculations with energies relative to the most stable isomer, including ZPE corrections. Panels (b) and (d) show dissociation energies for water evaporation from Cys(H_2_O)_6_^+^ and Cys(H_2_O)_6_H^+^, respectively. Horizontal lines above the parent cation represent ground state energy levels of the optimized parent cation isomers.

Fig. 5 presents the CID curves (relative ion yield as a function of the collision energy) for the main fragments obtained by colliding Cys(H_2_O)_6_^+^ (left) and Cys(H_2_O)_6_H^+^ (right) clusters with an Ar collision gas. The loss of the first molecule from the cluster requires only a small amount of activation energy below the detection limit of our experiments. Then a significant amount of energy is required for the evaporation of more water molecules from the cluster. By fitting the slope of the curves, we can obtain appearance energies for the ionic fragments resulting from the evaporation of 2, 3, 4, 5 or 6 water molecules, which are listed in Table 1. The differences between individual energies are similar for both protonated and nonprotonated clusters. This indicates that in both cases the ionized fragment is not water but rather cysteine. This is in good agreement with the fact that ionization potential of cysteine of 8.01 eV (the M06-2X/6-31++G(d,p) level of theory, present work) is lower than 12.6223 ± 0.0003 eV of water^84^ or that of the water dimer (11.21 ± 0.09 eV^85^). The structure with the charge on the cysteine moiety is confirmed by our quantum chemical modeling for both, Cys(H_2_O)_6_^+^ and Cys(H_2_O)_6_H^+^, with the most stable structures depicted in Fig. 6. We can see that in the case of Cys(H_2_O)_6_^+^ clusters, the water is accommodated around the carboxylic group, which has a higher proton affinity than the thiol group and, therefore, a proton is preferentially transferred from the thiol to the amino group. In the protonated form Cys(H_2_O)_6_H^+^, the only difference is the additional hydrogen on the thiol group. The charge remaining on the amino group in both cases and also the position of the water molecules is similar. The thiol thiolate change has a significant role in the biochemistry of cysteine, but we can see that in the present case of small water clusters it has practically no influence on the binding of water to the central cation. However, demonstrated by several recent theoretical and experimental studies of microhydrated nucleobases,^86–88^ the microhydrated environment does not necessarily resemble that of a bulk water.

**Table 1:** Appearance energies of ions resulting from fragmentation of Cys(H_2_O)_*n*_ and Cys(H_2_O)_*n*_H^+^ precursor cations

Precursor ion	Product ion [*m*/*z*]	*n*	Exp.	Calc.
Cys(H_2_O)_6_^+^	Cys.(H_2_O)_4_^+^ [193]	2	0.95 ± 0.1	0.94
	Cys.(H_2_O)_3_^+^ [175]	3	1.1 ± 0.1	1
	Cys.(H_2_O)_2_^+^ [157]	4	1.2 ± 0.3	1.1
	Cys.(H_2_O)^+^ [139]	5	1.4 ± 0.2	1.5
	Cys^+^ [121]	6	1.8 ± 0.15	1.8
	H_4_C_2_NO_2_^+^^74^	f	3.3 ± 0.6	4.21
	C_2_H_3_S^+ ^^59^	f	5.5 ± 1.7	6.03
Cys(H_2_O)_6_H^+^	Cys.(H_2_O)_4_H^+^ [194]	2	0.9 ± 0.1	0.95
	Cys.(H_2_O)_3_H^+^ [176]	3	1 ± 0.1	1.1
	Cys.(H_2_O)_2_H^+^ [158]	4	1.4 ± 0.3	1.2
	Cys.(H_2_O)H^+^ [140]	5	1.4 ± 0.2	1.5
	CysH^+^ [122]	6	1.9 ± 0.6	1.8
	H_6_C_2_NS^+ ^^76^	f	3.1 ± 0.2	3.24
	C_2_H_3_S^+ ^^59^	f	5.5 ± 0.7	5.90
Cys(H_2_O)_3_^+^	Cys(H_2_O)^+^ [139]	2	1.1 ± 0.7	1.1
	Cys^+^ [121]	3	1.4 ± 0.2	1.44
	H_4_C_2_NO_2_^+ ^^74^	f	2.9 ± 0.5	3.89
	C_2_H_3_S^+ ^^59^	f	4 ± 0.6	5.70
Cys(H_2_O)_3_H^+^	Cys(H_2_O)H^+^ [140]	2	1.1 ± 0.4	1.04
	CysH^+^ [122]	3	1.5 ± 0.5	1.4
	H_6_C_2_NS^+ ^^76^	f	3 ± 1.5	2.82
	C_2_H_3_S^+ ^^59^	f	5.0 ± 1.3	5.48

Calculations also give us an idea about the evaporation energies of water in the cluster cation ground state. They are listed in Table 1 together with estimates from the fits of the experimentally obtained CID curves. Generally the agreement is good.

It is important to say that the threshold values agree with the most energy efficient case, where neutral water is lost in the form of clusters and not in a sequence of individual molecules. The threshold energies for sequential loss are much higher as shown in the energy diagram in Fig. 6. In cluster studies, the evaporation of several molecular units is typically described as a sequential process.^89,90^ We cannot estimate the relative importance of the cluster *vs.* sequential water evaporation, but our data unambiguously show that water can also evaporate in the form of clusters. Such evaporation is then not restricted to the present system and activation process.

Table 1 also contains thresholds for fragmentation reactions of cysteine in the water environment. We can see good agreement of the theory and experiment for the protonated cluster cation reactions:Cys(H_2_O)_6_H^+^ → C_2_H_6_NS^+^ + CO + H_2_O + (H_2_O)_6_andCys(H_2_O)_6_H^+^ → C_2_H_3_S^+^ + CO + NH_3_ + H_2_O + (H_2_O)_6_

The fragmentation is in good agreement with a previous CID study of cysteine.^56^ However, we can see some disagreement between theory and experiment for the Cys(H_2_O)_6_^+^ clusters. In particular, the theoretical reaction thresholds for the reactions:Cys(H_2_O)_6_^+^ → H_4_C_2_NO_2_^+^ + CH_3_S + (H_2_O)_6_andCys(H_2_O)_6_^+^ → C_2_H_3_S^+^ + NH_3_ + HCO_2_ + (H_2_O)_6_are overestimated. There are several possible explanations for the discrepancy. *E.g.* the calculated reaction pathways or hydration sites of Cys(H_2_O)_6_^+^ cations and their fragments may not be such as observed in our calculations. Advanced dynamical modelling may help us to fully explore the energy and charge flow in these clusters prior to dissociation.(see *e.g.*^91^) An option remains the error of the used computational method. We already previously observed problems of the M06-2X functional in describing open shell species.^92^ In the following discussion of internal energy redistribution after collision-induced activation, we will therefore focus purely on the water evaporation channels, where the interpretation is clear.

### 3.3 Initial energy redistribution

What happens with the cluster when the collision energy is above the evaporation/fragmentation threshold? From the experimental data, we can see a very similar trend for most of the water evaporation channels. There is no significant competition between the channels. But does the process strictly reflect the collision energy? To probe this question we used advanced computational modelling using the M_3_C method. Let us mention that this is the first time this complex method has been used for molecular clusters of this size.

**Fig. 7:**
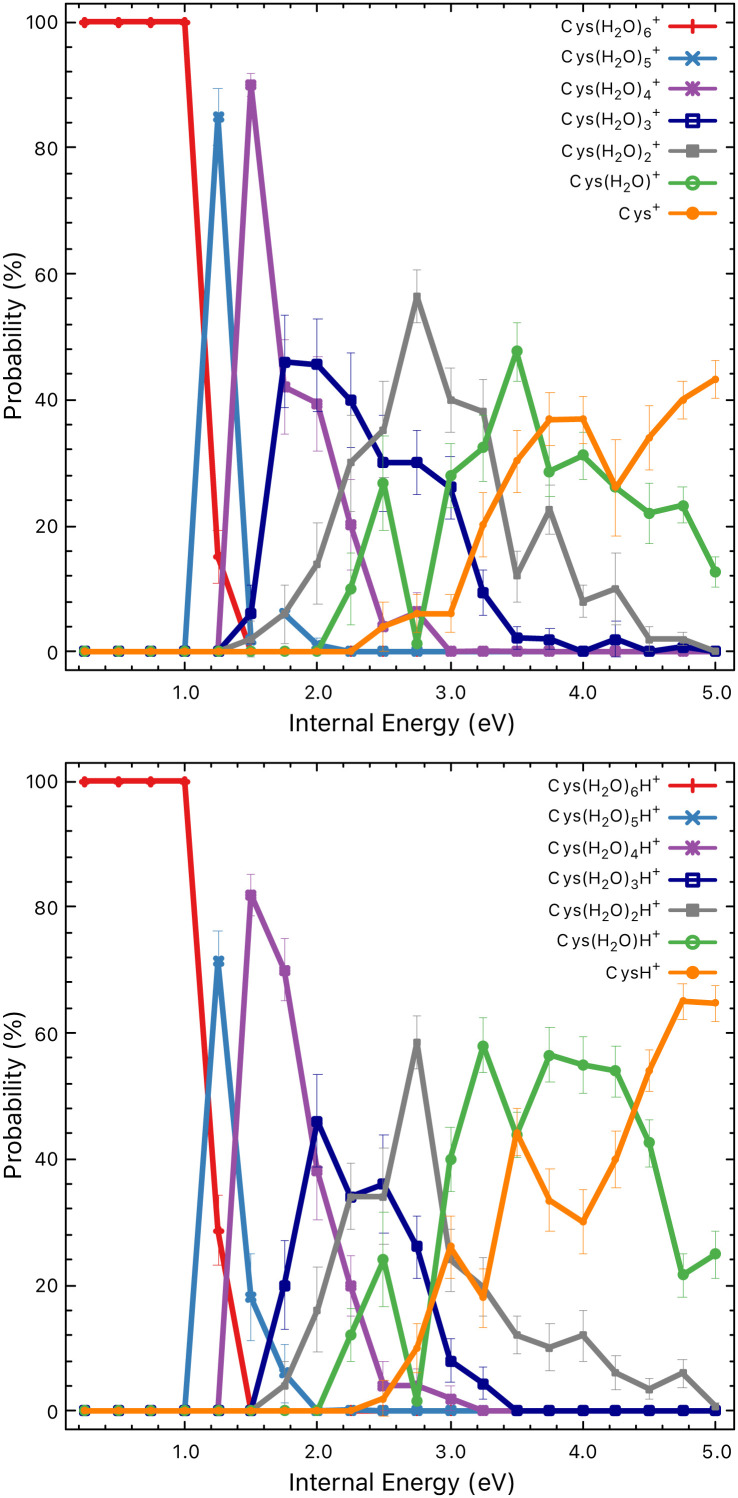
Probabilities of particular charged fragments as a function of the internal energy of the Cys(H_2_O)_6_^+^ (top) and Cys(H_2_O)_6_H^+^ (bottom) cluster cations calculated using the M_3_C approach.

First, in Fig. 7, we can see the probability of producing charged fragments at different internal energies of the cluster cations. We can understand the figures in the following way: if the internal energy is 0.5 eV then there should be no fragmentation, at 1.5 eV the fragmentation pattern should be dominated by the loss of two water molecules with minor contributions from one or three water losses and so on. Comparing the model to the experimental data shown in Fig. 5, we can see that the probability of the fragmentation observed in the experiment is much lower than the one predicted by the model. This is caused mainly by the fact that, in CID experiments, precursor ions are detected with higher intensities. Therefore, the precursor ion intensities are excluded from the further analysis.

**Fig. 8:**
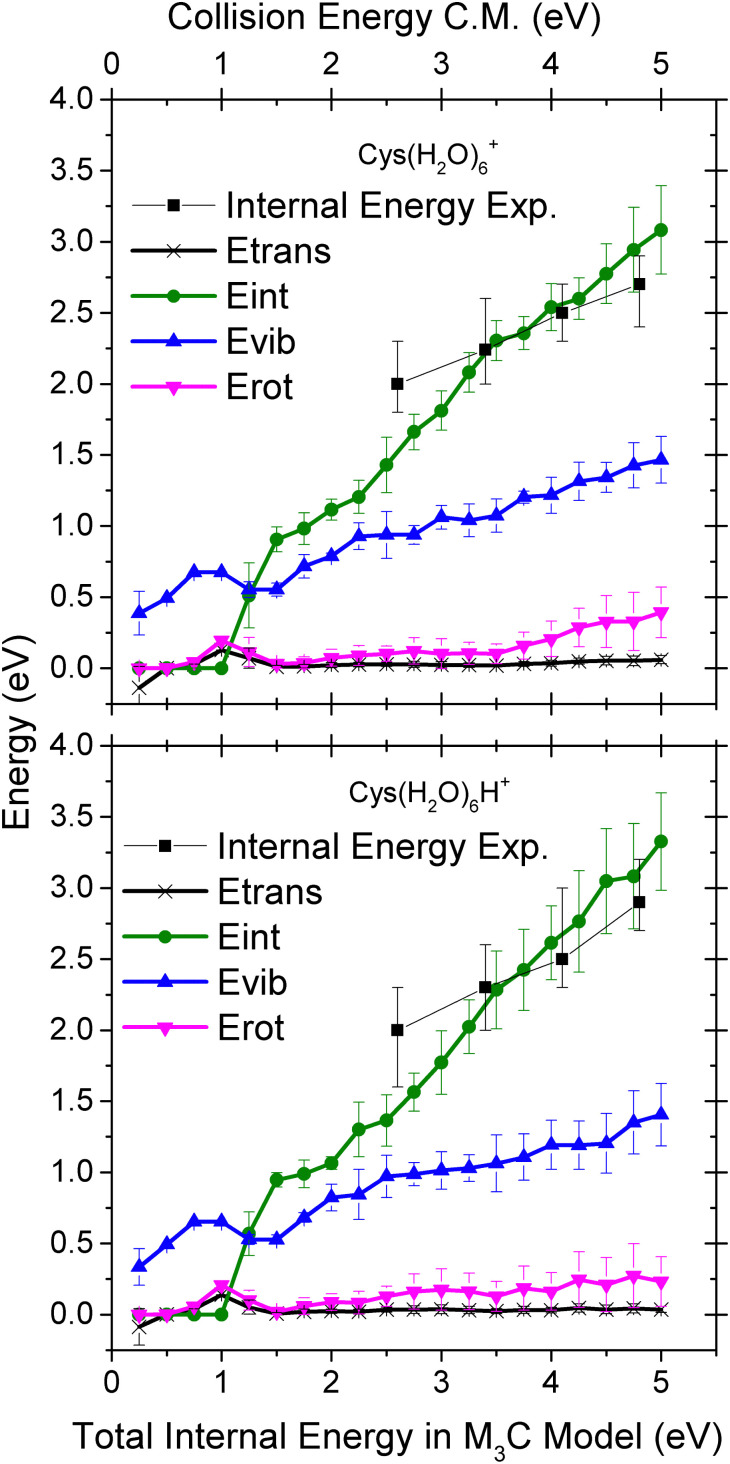
Distribution of the energy transferred to the cluster degrees of freedom upon collision-induced activation as a function of the internal energy of the Cys(H_2_O)_6_^+^ (top) and Cys(H_2_O)_6_H^+^ (bottom) cluster cations calculated using the M_3_C approach. The black squares represent the mean value of the internal energy distributions obtained by fitting the experimental mass spectra (see Fig. 9).

In Fig. 8 we show theoretical data from the M_3_C model with the redistribution of the internal energy of the system among its different components: translational, vibrational, rotational and intermolecular. Intermolecular energy corresponds to the sum of the electronic energies of the fragments (relative to the parent ion) in a given fragmentation channel. We can see that this energy is non-zero when the first fragmentation channel is opened at an internal energy of ∼1 eV. The translational energy remains low and nearly constant throughout the entire simulation, whereas with the increasing internal energy the vibrational component increases.

**Fig. 9:**
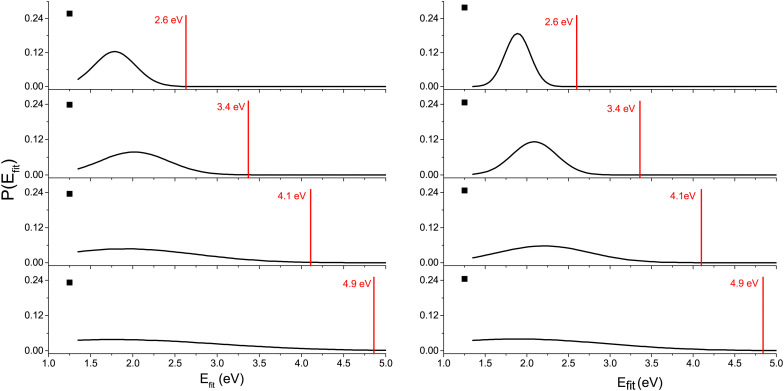
Internal energy distribution after collision-induced activation of Cys(H_2_O)_6_^+^ (left) and Cys(H_2_O)_6_H^+^ (right) clusters based on fits of the experimentally obtained mass spectra by fragmentation probabilities at different internal energies obtained in the M_3_C calculations (see the ESI† for details). The first water molecule is efficiently ejected *via* a non-ergodic process, which is described by a discrete point in the distribution (black square). Red vertical lines indicate the collision energy in the center of mass frame.

To deduce the internal energy of the clusters after collision-induced activation, we use the modelled M_3_C fragmentation patterns at individual energies, given in Fig. 7, to fit our experimentally obtained fragmentation patterns in Fig. 5. Details of the fitting procedure can be found in the ESI.† We focus purely on the evaporation of water. This approach converts the center of mass collision energy to the internal energy of the cluster under the assumption that fragmentation occurs according to the M_3_C model. The results of the fit are shown in Fig. 9. Note that clusters included in the M_3_C simulations are stable structures, local minima in the potential energy surface. We thus assume that metastable states that might be created shall evolve towards local minima with the excess of excitation energy redistributed among the nuclear degrees of freedom.

In every internal energy distribution of Fig. 9 obtained from the fit to experimental fragmentation patterns, we see a single Gaussian peak, which correlates well the fragmentation pattern to the M_3_C model, but at an energy lower than the collision energy. Then we can see a single discrete point at 1.25 eV, which was required to account for the high intensity of the single molecule evaporation channel. The most probable energy obtained as a mean of the peak at higher energy can be used for comparison with the M_3_C model. These most probable values are shown as black squares in the plots of Fig. 8. The values are approximately two thirds of the collision energy. However, slopes of the dependencies around 0.35 indicate that at higher collision energies, even less energy will be redistributed according to the statistical M_3_C model.

The fact that not all the energy available in the collision process is resulting in ergodic fragmentation of the parent cluster cation is not surprising in the view of previous CID studies of weakly bonded clusters (*e.g.*ref. 18). In the present case, the discussion of the basic assumptions of the M_3_C model enable us to better understand this difference.

First, while modelling the experiment, we neglect molecular fragmentation. Since the explored collision energies are smaller than 5 eV and below the calculated and detected molecular dissociation thresholds, we believe this assumption cannot lead to a significant difference in the observed energy transfer.

Another important fact that has been neglected is the initial internal energy of the precursor cluster cations that can result in a spontaneous decay *via* loss of a single water molecule. A possible explanation is that part of our cluster distribution is metastable with respect to evaporation of a single molecular unit. This is consistent with the high intensity of the single water molecule loss channel, fitted by a single discrete point in the energy distributions in Fig. 9. The yield of cations with one water molecule less may be caused by the decay of metastable cluster ions excited during the extraction from the He droplets.^66^ Several observations indicate that such an explanation cannot be used in the present case. First, parameters of the cluster ion extraction from He droplets were set to see several He taggants to the cluster ions (see ESI†), which means the temperature of the ions has to be low, below the He binding energy. Second, metastable decay does not depend on the collision energy. However, the energy-dependent ion yield curve corresponding to the single molecule loss (Fig. 5) varies with energy. Particularly, one can see clear competition with other fragmentation channels (note the log scale of the *y* axis). Third, if the clusters are hot prior to the collision-induced activation, we will see increased single molecule loss but the rest of the fragmentation pattern will correspond to the M_3_C predicted fragmentation, exactly at the collision energy. In other words, the single loss channel will be a result of a surplus of internal energy, which we do not observe. Rather, the collision energy is partitioned between the single molecule loss channel and fragmentation according to the M_3_C model.

The discussion leads us to the most important assumption of the M_3_C model, which is ergodic redistribution of internal energy. While in many molecular systems^79^ and ionization or excitation events^69^ this assumption is valid, it does not seem to be valid in the present case. The high yield of single molecule evaporation events actually indicates a non-ergodic process, in which a single water molecule can be ejected from the cluster. In such a process, a significant portion of the collision energy can be taken away in the form of kinetic energy of the ejected water molecule. Indeed, this mechanism was observed for high energy collisions of small protonated water clusters with Ar.^93^ More recently, “impulsive” dissociation was used to describe uracil–water cluster ion dissociation upon collisions with 7.2 eV Ne atoms indicating rather common occurrence of this mechanism in the collision activation process.^87,94^ We conclude that non-ergodic loss of a single water molecule represents the most plausible explanation of the high intensity of this reaction channel in the present experiment. Since the “ergodic” peak in the fitted energy distributions (Fig. 9) is shifted towards lower energy with respect to collision energy, we conclude that the collision energy can be distributed between the non-ergodic release of a fast water fragment and ergodic increase in the internal energy during the single collision event. Full understanding of the process can be obtained studying larger cluster systems and using different experimental (*e.g.*^93^) and theoretical methods (*e.g.*^94,95^), going beyond the scope of the present paper.

## 4 Conclusions

We prepared Cys(H_2_O)_6_^+^ and Cys(H_2_O)_6_H^+^ ions by assembling inside He droplets suppressing the clustering of cysteine. After activation, the ions primarily fragment *via* the loss of water molecules. No proton transfer, which is typical for heterogeneous clusters of amino acids, was observed.

We report appearance energies for water evaporation and some fragmentation reactions estimated from the CID data in good agreement with values from M06-2X/6-31++G(d,p) calculations. Comparison of the data demonstrates that water solvated clusters can fragment *via* evaporation of neutral water clusters.

For the first time, we used the M_3_C method to estimate the initial energy transferred to a cluster by collisional activation. Direct comparison with the experiment demonstrates that only about 2/3 of the collision energy is redistributed according to the ergodic hypothesis and the rest is taken away by the first molecule evaporated from the cluster. Such a fragmentation, where a single molecule of weakly bound clusters takes away a significant amount of the collision energy can be common and particularly important in analytical chemistry using electrospray or proton transfer ionization coupled with tandem mass spectrometry and has to be taken into account, particularly when extracting kinetic parameters.

## Conflicts of interest

There are no conflicts to declare.

## Supplementary Material
